# Retraction: Curcumin Exerts Effects on the Pathophysiology of Alzheimer's Disease by Regulating PI(3,5)P2 and Transient Receptor Potential Mucolipin-1 Expression

**DOI:** 10.3389/fneur.2019.00255

**Published:** 2019-02-27

**Authors:** Frontiers Editorial Office

The Journal and Chief Editors retract the October 2017 article cited above.

Following the publication of the article, concerns with the study's methodology were addressed to the Journal and its Editorial Board. Despite extensive efforts to clarify these with the authors and their institution, we have not been able to obtain a response. Therefore, following further independent assessment of the concerns raised, the Journal retracts the article, without the authors' agreement, due to these concerns.

